# Endothelium-targeted human Delta-like 1 enhances the regeneration and homing of human cord blood stem and progenitor cells

**DOI:** 10.1186/s12967-015-0761-0

**Published:** 2016-01-06

**Authors:** Deng-Mei Tian, Ying-Min Liang, Yong-Qing Zhang

**Affiliations:** Department of Hematology, 309th Hospital, Chinese People’s Liberation Army, Hei-san hu Street #17, 100091 Beijing, China; Department of Hematology, Tangdu Hospital, Fourth Military Medical University, Xi’an, China

**Keywords:** HSPCs, Notch, Delta-like 1, Regeneration, Homing

## Abstract

**Background:**

Umbilical cord blood (UCB) is becoming an alternative cell source for hematopoietic stem cell transplantation (HSCT). However, umbilical cord blood transplantation (UCBT) has been severely limited by low and finite numbers of hematopoietic stem cells and their delayed engraftment. New strategies are needed to improve ex vivo expansion efficiency and in vivo haematopoietic recovery.

**Methods:**

We produced an endothelium-targeted soluble Notch ligand, the Delta-Serrate-Lag-2 (DSL) domain of human Delta-like 1 fused with a RGD motif (hD1R), and tested the effects of this protein on human umbilical cord blood hematopoietic stem and progenitor cell (UCB-HSPC) ex vivo and in vivo.

**Results:**

hD1R-mediated ex vivo expansion system was able to significantly increase the absolute number of UCB-HSPCs. The hD1R-expanded cells had the enhanced homing and maintained long-term hematopoietic stem cell repopulation capacity in the bone marrow of immunodeficient nonobese diabetic-severe combined immunodeficient (NOD/SCID) mice. Moreover, systemic administration of hD1R promoted the in vivo regeneration of donor cells in recipient mice and accelerated hematopoietic recovery, particularly in settings wherein the HSPCs dose was limiting.

**Conclusions:**

Our results indicated that hD1R might be applied in improving hematopoietic recovery and HSC engraftment in human UCBT.

**Electronic supplementary material:**

The online version of this article (doi:10.1186/s12967-015-0761-0) contains supplementary material, which is available to authorized users.

## Background

Allogeneic hematopoietic stem cell transplantation (Allo-HSCT) is recognized as an effective treatment for patients with hematological and nonhematological malignancies. Umbilical cord blood (UCB) is becoming an alternative source of hematopoietic stem cells for patients requiring allo-HSCT when no suitable donors are available [1]. Its rapid availability and tolerance to higher HLA disparity have more advantage than bone marrow (BM) or peripheral-blood (PB) stem cell transplantation. Despite these, the clinical application of UCB in the adults has been restricted by the low and finite hematopoietic stem and progenitors cell (HSPC) dose, which resulted in delayed engraftment and increased rates of infectious complications [2, 3]. Several strategies were explored to ameliorate outcomes after UCB transplantation (UCBT), including infusion of double UCB units (DUCB), ex vivo expansion of HSCs and so on [4, 5]. Although DUCB increased HSPC numbers and promoted engraftment rates, the risk of GVHD (graft versus host disease) significantly rose [6]. In addition, multiple cytokines were used to expand HSPCs ex vivo in UCB, but few increased cells achieved long-term engraftment, suggesting expansion of hematopoietic progenitor cells (HPCs) [7, 8].

HSCs are normally accommodated in stem cell niches, where specific niche cells are thought to provide the necessary paracrine factors and direct cell–cell interaction to maintain the proliferation and differentiation of HSCs [9–11]. Many studies investigated the expansion of HSCs on mesenchymal progenitor, endothelial cell (EC) feeder layers, and CD146^+^ pericytes, which were used as surrogates for in vivo stem cell niches and provided necessary signaling cues to maintain the stemness of expanded cells [12–14]. However, due to difference in the cytokines used and loss of long-term engraftable cells in vitro expansion, an effective protocol for the expansion of UCB HSCs is still to be explored.

The Notch signaling pathway plays a key role in regulating the proliferation and differentiation of stem cells [15]. In mammals, the canonical Notch pathway includes five Notch ligands (Delta-like [Dll] 1, 3, 4, Jagged 1, 2), four Notch receptors (Notch1–4), the transcription factor RBP-J, and the downstream effectors such as the Hairy and enhancer of split (Hes) family molecules [16]. Early work showed that Notch receptors and ligands were expressed by BM stromal and hematopoietic cells [17, 18]. Targeted disruption of Notch pathway diminished stemness of HSCs. Several reports showed that Notch 1, Dll-1, and Jagged-1 could induce expansion of HSPCs, and that ligand immobilization was required to activate Notch signaling [19–22]. Other studies demonstrated the selectivity and dose-dependency of ligands in HSPCs expansion ex vivo [23]. Furthermore, Delaney et al. [24] reported that the average time of neutropenia was significantly shortened in patients who received human UCB CD34^+^ cells expanded with the immobilized Delta 1 in a phase I clinical trial of transplantation.

Our previous research demonstrated endothelium-targeted Delta-like 1 (D1R) significantly promoted the expansion of mouse and human HSPCs. Moreover, mouse D1R (mD1R) could directly stimulate hematopoiesis in vivo in normal or sublethally irradiated mice. mD1R might promote HSCs engraftment through influencing, structurally and/or functionally, stem cell niches [25]. However, the in vivo roles of human D1R (hD1R) on hematologic recovery and the engraftment of donor cells in HSCT were not explored in detail. In the present study, we purified hD1R fusion protein, which was composed of the DSL domain of the human Dll1 and a RGD (arginine–glycine–aspartic acid) motif binding to the endothelial integrin αvβ3 and triggering ligand endocytosis. We demonstrated that hD1R promoted the regeneration and homing of human UCB HSPCs and enhanced HSC engraftment in vivo in transplantation.

## Methods

### DNA recombination and production of recombinant proteins

The cDNA fragment encoding the DSL domain (amino acids 127–225) of the human Dll1 (NM_005618) was amplified by PCR using a human cDNA library as a template. The product was fused at the C-terminus with a RGD-encoding fragment (hD1R) by PCR. The resultant gene fragments were cloned into pET32a (+) between the *Nco* I and *Xho* I sites, to construct pET32a-hD1R. For the production of recombinant proteins,* E. coli* BL21 (DE3) were transformed with the plasmids. Positive clones were expanded in Luria–Bertani (LB) medium, and cells at the exponential stage were induced with 0.5 mM isopropyl β-D-thiogalactoside (IPTG). The Trx-tagged proteins were purified by using Ni2^+^-NTA columns (Invitrogen, Carlsbad, CA) according to the manufacturer’s manual. To obtain the S-tagged proteins, Trx-hD1R were cleaved by using thrombin (Novagen, Darmstadt, Germany), and further purified using Ni2^+^-NTA columns. The hD1R protein was prepared in the Department of Medical Genetics and Developmental Biology of Fourth Military Medical University and has been detailed previously [25, 26].

### Cell culture

Human umbilical vein endothelial cells (HUVECs) were cultured in M199 medium (GIBCO, Gaithersburg, MD) supplemented with 20 % fetal bovine serum (FBS), 30 μg/mL endothelial cell growth supplement (ECGS) (Sigma, St Louis, MO), 20 units/mL heparin, 100 U/mL penicillin, and 100 μg/mL streptomycin. Cells between passage three and five were used for experiments. For co-culture, HUVECs (2 × 10^4^) were seeded in wells of 24-well plates and cultured to confluence. Cells were treated with mitomycin C (10 μg/mL) for 2.5 h, and were washed with PBS thoroughly for three times. Human UCB CD34^+^ progenitor cells were purified from human UCB samples by FACS-sorting after being stained with anti-human CD34-FITC (#581, Biolegend). The cells (2 × 10^3^) were then plated on HUVECs and cultured in serum-free medium (StemSpan SFEM, STEMCELL Technologies, Vancouver, Canada) supplemented with a cocktail containing five types of human cytokines (h5GF) including thrombopoietin (TPO, 20 ng/mL), stem cell factor (SCF, 120 ng/mL), Flt-3 ligand (Flt-3L, 50 ng/mL), interleukin 6 (IL-6, 5 ng/mL), and interleukin 3 (IL-3, 5 ng/mL) (PeproTech, Rocky Hill, NJ). hD1R was added at the concentration of 2.5 μg/mL as previously described [25]. In some experiments, γ-secretase inhibitor (GSI) (DAPT, Alexis Biochemicals, San Diego, CA) was included at the concentration of 10 μM. Half amount of the medium was changed every other day. Seven days after the starting of the co-culture, cells in suspension were collected by gentle pipetting and analyzed further. In some experiments, confluent HUVECs were cultured for 48 h in serum-free medium and supernatant containing soluble factor were collected and filtered through a 0.22 μm sterile filter as culture conditioned media. Live HUVECs were fixed 4 % paraformaldehyde (PFA) for 15 min and then used for co-culture experiments. Experiments associated with human samples were approved by the Ethical Committee on Medical Research-Related Affairs of the Fourth Military Medical University.

### Colony-forming units (CFU) assay

CFU assay was performed by mixing freshly isolated or cultured hematopoietic cells with Methocult GF H4434 medium (STEMCELL Technologies). Cells were cultured for 14 days, and colonies (with >50 cells) containing different lineages of cells were counted under a microscope.

### Flow cytometry

FACS analysis was performed routinely by using a CaliburTM flow cytometer (BD Immunocytometry Systems). Anti-mouse CD45-FITC (#104, eBioscience), anti-human CD45-APC (HI30, eBioscience), anti-human CD34-FITC (#581, Biolegend). Cell-cycle analysis was performed using DNA binding dye propidiumiodide (PI). Hematopoietic cells were fixed in 50 % ethanol and resuspended to 0.2 mL of 10 mg/mL RNAaseA and 50 µg/mL PI. Cell-cycle kinetics was performed with routine protocols using the FACS Calibur flow cytometer (Becton–Dickinson, CA). Apoptosis was analyzed by using an Annexin V-FITC Apoptosis Detection Kit (4A Biotech, Beijing, China).

### Real time reverse transcription-polymerase chain reaction (RT-PCR)

Total RNA was extracted by using the Trizol reagent (Invitrogen). cDNA was prepared by using a kit from TOYOBO (Osaka, Japan) with random primers. Real time PCR was performed by using a kit (SYBR Premix EX Taq, Takara) and the ABI Prism 7500 real time PCR system, with β-actin as a reference control. Primers used in RT-PCR were as follows: β-actin-F: 5′-TGGCACCCAGCACAATGAA; β-actin-R: 5′-CTAAGTCATAGTCCGCCTAGAAGCA; CXCR4-F: 5′-CCTATGCAAGGCAGTCCATGT; CXCR4-R: 5′-CTAAGTCATAGTCCGCCTAGAAGCA; Hes1-F: 5′-TGGAAATGACAGTGAAGCACCTC; Hes1-R: 5′-TCGTTCATGCACTCGCTGAAG; α4integrin-F: 5′-GGAATATCCAGTTTTTACACAAAGG; α4integrin-R: 5′-AGAGAGCCAGTCCAGTAAGATGA; α6integrin-F: 5′-ATGCACGCGGATCGAGTTT; α6integrin-R: 5′-TTCCTGCTTCGTATTAACATGCT.

### NOD/SCID transplantation

NOD/SCID mice of 6–8 weeks old were purchased from Beijing HFK Bioscience Co. Ltd and were maintained in axenic conditions and sublethally (300 cGy) irradiated by total-body irradiation with γ-ray from a ^60^Co irradiation apparatus. Freshly isolated BM cells or in vitro expanded cells were infused via the tail vein. Mice were then maintained with gentamycin sulfate-containing water until further experiments. The homing efficiency of the transplanted cells was estimated by flow cytometry. For limit dilution assays, serial numbers of uncultured CD34^+^ UCB-derived cells or a fraction of the final culture equivalent to starting cells (10^3^, 6 × 10^3^, 10 × 10^3^ cells) were transplanted. Positively engrafted mice were regarded as to have ≥0.5 % of human CD45^+^ (hCD45^+^) cell in peripheral blood 12 weeks after the transplantation. The percentage of the negatively engrafted mice was plotted against the initial numbers of cells infused. SCID-repopulating cell (SRC) frequency was estimated by using the L-Calc software (STEMCELL Technologies) as the cell number by which 37 % of the mice were non-engrafted. For secondary engraftment, 50 % of the bone marrow (from both femurs and tibiae) from each recipient mouse (10 × 10^3^ cells group) was transplanted into one secondary sublethally irradiated mouse. 12 weeks after transplantation, bone marrow was harvested from the secondary mice and analyzed by flow cytometry. All animal experiments were approved by the Animal Experiment Administration Committee of the Fourth Military Medical University.

### Migration assays

Chemotaxis experiments were performed in polycarbonate transwell inserts (5-mm pore, Corning Costar Corp.). Migration medium with 100 ng/mL SDF-1 (Peprotech) was added in the lower chamber. Freshly purified CD34^+^ cells or cells expanded ex vivo (2 × 10^5^) were seeded in the upper compartment. After 4 h of incubation at 37 °C, migrated cells in the lower chamber were enumerated by flow cytometry. The percentage of migration was calculated by dividing total cells migrated to the lower well by the cell input multiplied by 100.

### Peripheral blood analysis

Retro-orbital peripheral blood was collected using microhematocrit capillary tubes (Sanger Biotech, China). Differential blood counts were obtained using PE-6000 Hematology Analyzer (Prokan Electronics Inc., China).

### Histology

Tissues were fixed in 4 % paraformaldehyde overnight. Femurs were decalcified for 21 days using an EDTA decalcifying solution for paraffin section. H&E staining of bone sections were performed using standard procedures. Images were taken under a fluorescence microscope (Olympus BX51, Japan).

### Statistics

Data were analyzed with the SPSS 17.0 software. Comparisons between groups were undertaken by using the unpaired Students’ *t* test. Results were expressed as the mean ± standard deviation (SD). *P* < 0.05 was considered statistically significant.

## Results

### Direct cellular contact with HUVECs improved hD1R-induced human UCB HSPCs expansion ex vivo

**Fig. 1:**
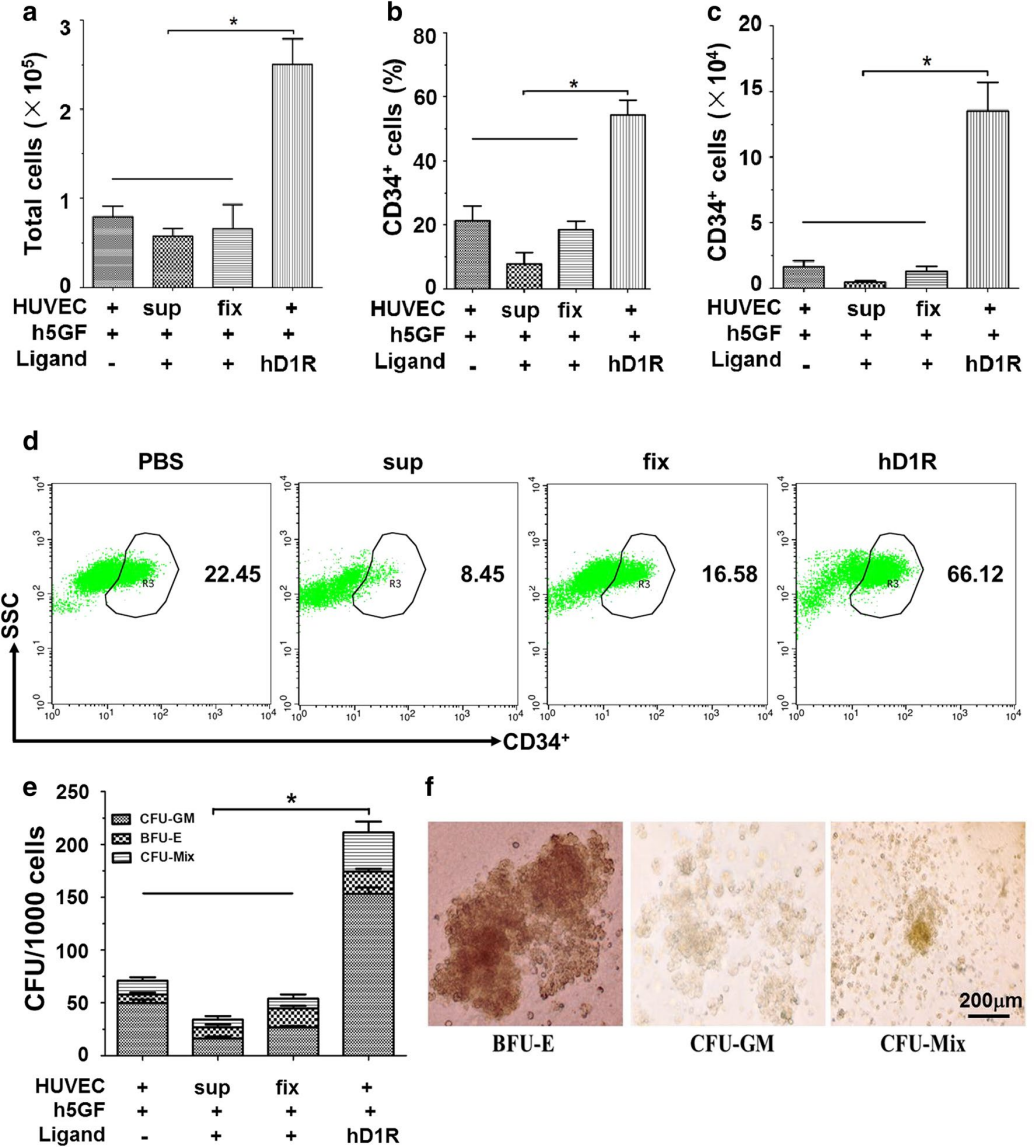
Human UCB CD34^+^ cells expansion by hD1R requires direct cellular interaction with live HUVECs. **a** CD34^+^ cells (2 × 10^3^) were cultured with or without HUVECs in the presence of h5GF plus hD1R. The cells were harvested on day 7 and counted (n = 4). **b**, **c** The cells in (**a**) were analyzed by FACS to determine the percentage (**b**) and the number (**c**) of CD34^+^ cells (n = 4). **d** Representative FACS analysis of CD34^+^ cells in (**b**). **e** The cells (1 × 10^3^) in (**a**) were used for colony-forming assay (n = 4). **f** Photomicrographs of human hemopoietic cell colonies. *Scale bar* represented 200 μm. Bars = mean ± SD. *P < 0.05

We purified a fusion protein composed of the DSL domain of human Dll1 and a RGD motif (hD1R) as previously described [25, 26] (Additional file 1: Figure S1A, 1B). To assess the effects of hD1R on UCB HSPCs, we tried to expand human CD34^+^ cells ex vivo under different culture conditions. It should be noted that different conditions were tested: (1) Co-culture with HUVECs supplemented with h5GF in the presence of PBS or hD1R; (2) Supernatant from HUVECs (sup group) in the presence of h5GF plus hD1R; (3) Fixed HUVECs by 4 % PFA (fix group) with h5GF plus hD1R. Human cord blood CD34^+^ cells (2 × 10^3^) were cultured under different conditions. After 7 days in culture, we harvested cells and measured the expression of Hes1 by quantitative RT-PCR. hD1R up-regulated Hes1 expression in the hematopoietic cells in the co-culture with HUVECs + h5GF compared to other groups. Moreover, GSI effectively blocked Hes1 expression (Additional file 1: Figure S1C). These results suggested that live HUVECs were necessary for hD1R to activate Notch signaling. Next, we observed that hD1R had the effect on CD34^+^ cells in different culture conditions. Seven days after the starting of the co-culture, we found that total cell numbers significantly increased in the culture with HUVECs supplemented with h5GF in the presence of hD1R, resulting in a fold expansion of 123.5 ± 3.9 nucleated cells (Fig. 1a; Additional file 1: Figure S2). FACS analysis showed that the percentage of CD34^+^ cells increased significantly in the culture with HUVECs plus hD1R plus h5GF (Fig. 1b, d), and the number of CD34^+^ cells was expanded 87.5 ± 2.5-folds. Supernatant containing soluble angiocrine factors from HUVECs failed to efficiently expand CD34^+^ cells and accelerated CD34^+^ cells differentiation (Fig. 1c, d). Similarly, on co-culture with fixed HUVECs, total cells number and CD34^+^ cells number significantly decreased. The expanded cells from each group were subjected to CFU assay. The results showed that the total number of colonies and the numbers of burst-forming unit-erythroid (BFU-E), CFU-granulocyte macrophage (CFU-GM), and CFU-granulocyte, erythrocyte, monocyte, macrophage (CFU-GEMM) significantly increased in the co-culture with HUVECs in the presence of hD1R plus h5GF (Fig. 1e, f). Thus, these data showed that expansion of HSPCs in the presence of hD1R required live HUVECs and direct cell–cell contacts between hematopoietic cells and HUVECs.

**Fig. 2:**
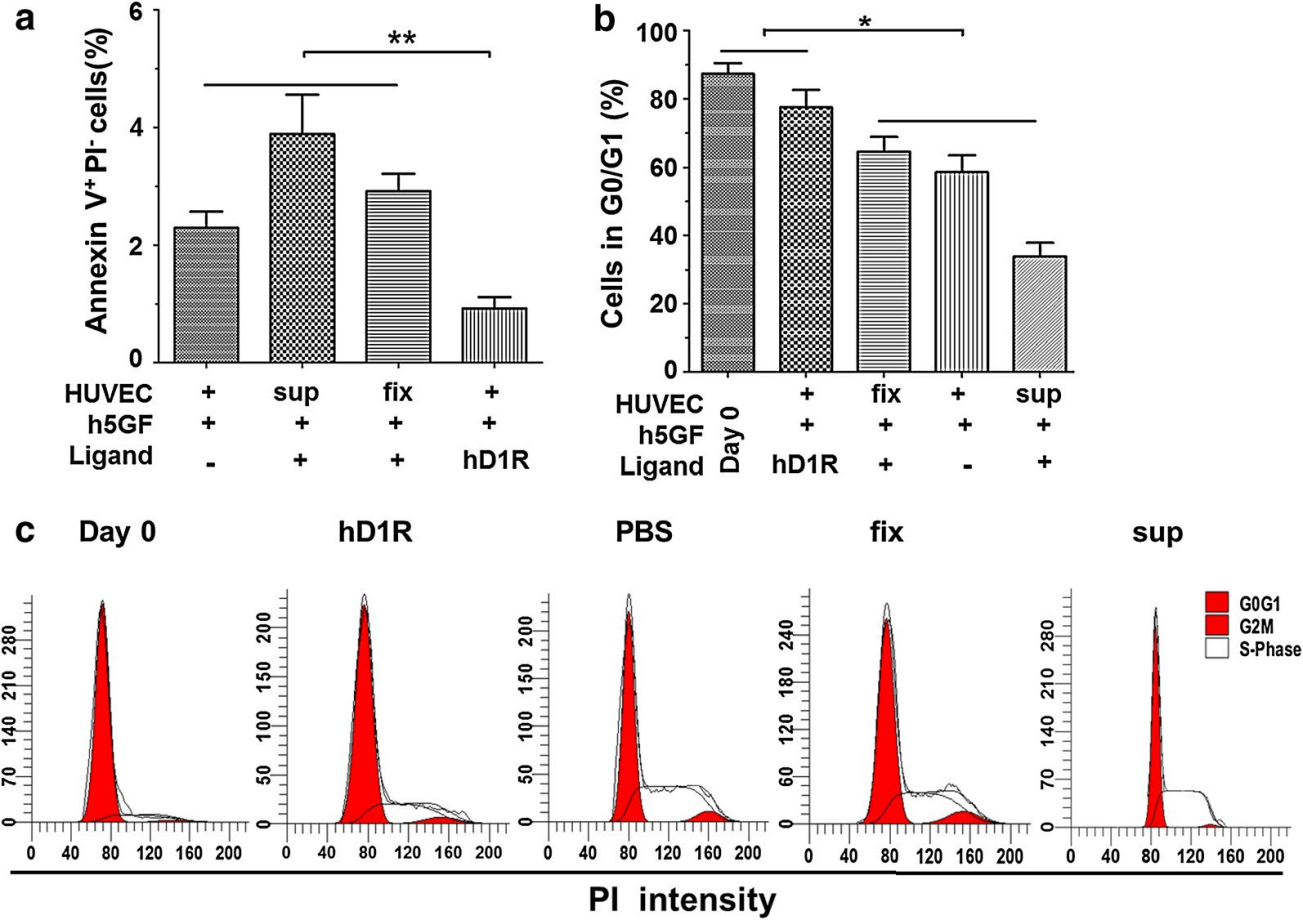
hD1R maintained quiescence and regeneration of human UCB CD34^+^ cells. CD34^+^ cells were cultured with or without HUVECs in the presence of h5GF plus hD1R. The cells were harvested on day 7. **a** Cultured cells were analyzed by using annexin V staining followed by FACS to determine the percentage of cell apoptosis (n = 4). **b** Cell-cycle analysis of CD34^+^ cells expanded in different conditions or freshly purified from UCB (Day 0). The number of cells in various stages of the cell cycle was quantified measuring the area under the red peaks (G0/G1). **c** Representative plots of cell-cycle analysis of CD34^+^ cells in (**c**). Bars = mean ± SD. *P < 0.05

We analyzed cell apoptosis in the ex vivo culture by using annexin V staining followed by FACS. The data showed that a onefold less portion of CD34^+^ underwent apoptosis (defined as annexin V positive, propidium iodide negative CD34^+^ cells by FACS) in the co-culture with HUVECs in the presence of hD1R. However, co-culture without HUVECs or with fixed HUVECs promoted cell apoptosis (Fig. 2a; Additional file 1: Figure S3). These data suggested that hD1R provided a survival signal and inhibited apoptosis in the co-culture with live HUVECs. Similarly, cell-cycle analysis indicated CD34^+^ cells expanded on HUVECs with hD1R + h5GF maintained higher G0/G1 phases than those co-cultured without HUVECs or with fixed HUVECs (Fig. 2b, c), which prevented exhaustion of cycling CD34^+^ cells.

### hD1R promoted long-term repopulating capacity of HSPCs

**Fig. 3:**
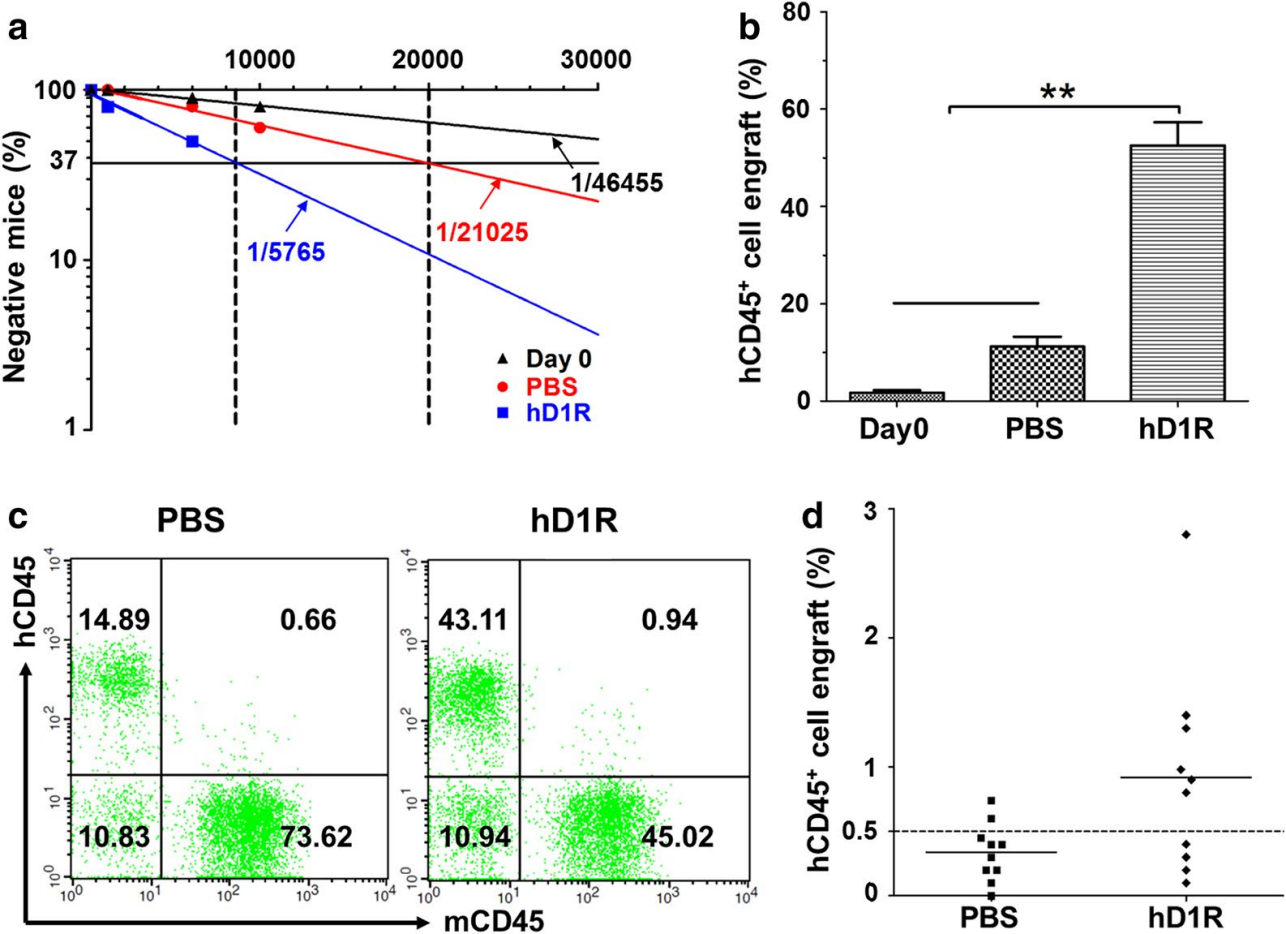
Repopulation of sublethally irradiated mice with expanded human CD34^+^ cells. **a** SRC assay. CD34^+^ cells freshly purified from UCB (Day 0) (10^3^, 6 × 10^3^, 10 × 10^3^ starting cells) or their progeny expanded with HUVECs + h5GF plus PBS or hD1R were transplanted into sublethally irradiated NOD/SCID mice. The peripheral blood cells of the recipients were analyzed by FACS 12 weeks post transplantation. The number of transplanted cells was plotted against the percentage of mice without engraftment to determine the frequency of repopulating units (n = 30). **b**, **c** The cells in (**a**) were transfused into sublethally irradiated NOD-SCID mice. In the 10 × 10^3^ cells group, the peripheral blood cells of the recipients were analyzed by FACS 12 weeks post transplantation. The percentages of engrafted human CD45^+^ were compared (**b**) (n = 10). **c** Representative FACS analysis of hCD45^+^ cell engraftment in (**b**). **d** Human CD45^+^ cell engraftment 12 weeks after the secondary transplantation for primary recipient mice in the 10 × 10^3^ cells group. *Horizontal bars* represented mean levels of hCD45^+^ cell engraftment in BM. (n = 10). Bars = mean ± SD. *P < 0.05

To assess functional HSCs expanded by hD1R, we quantified SRC frequency by limiting-dilution analysis. Different numbers (10^3^, 6 × 10^3^, 10 × 10^3^ starting cells) of freshly isolated CD34^+^ cells (Day 0) or their progeny expanded on HUVECs + h5GF in the presence of PBS or hD1R were transplanted into sublethally irradiated NOD/SCID mice. The percentages of hCD45^+^ cells in peripheral blood were determined by FACS 12 weeks later. The results showed a 3.6-folds higher of the frequency of repopulating cells in the hD1R-expanded population (1/5765, with a 95 % confidence interval from 1/7805 to 1/4258) than that in the PBS group (1/21025, with a 95 % confidence interval from 1/32613 to 1/13555), and a 8.1-folds increase compared to that in the Day 0 group (1/46455, with a 95 % confidence interval from 1/86190 to 1/25039) (Fig. 3a). This represented a total SRC content in three independent experiments (Additional file 1: Table S1). In the 10 × 10^3^ cell groups, the cells cultured in the presence of hD1R exhibited significantly higher level of engraftment than the cells of the control (Fig. 3b, c). To confirm that hD1R expanded HSCs with serial repopulating capacity, we performed a second engraftment experiment for the recipient mice in the 10 × 10^3^ cells group. We infused 50 % bone marrow of primary recipients of hD1R-expanded population or PBS-expanded ones into secondary NOD/SCID mice. The percentages of engrafted mice (with ≥0.5 % hCD45^+^ cells in BM) were analyzed by FACS 12 weeks post transplantation. The mean engraftment level in the recipients of hD1R-expanded group was higher than that in the PBS-expanded one (Fig. 3d). These data indicated that hD1R with HUVECs plus h5GF promoted a substantial increase of long-term repopulating HSCs in culture.

### hD1R enhanced HSC homing to the marrow

**Fig. 4:**
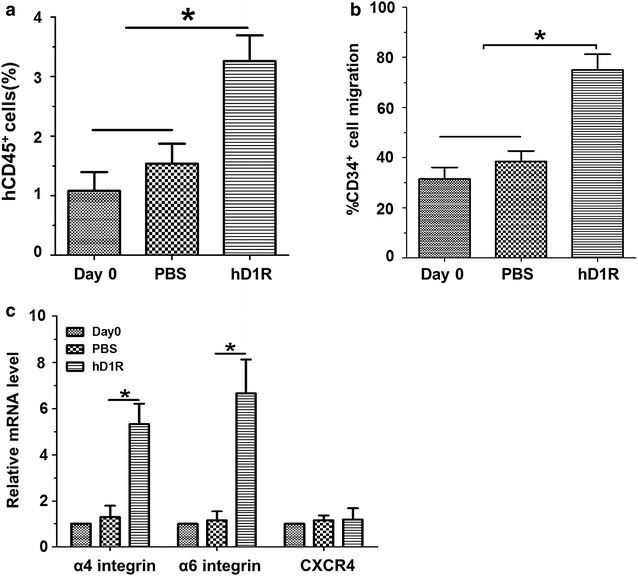
hD1R enhanced the homing efficiency of CD34^+^ cells. **a** Homing efficiency. CD34^+^ cells (2 × 10^5^) were purified from UCB (Day 0) or cells expanded ex vivo transplanted into sublethally irradiated mice. BM cells from the recipients were analyzed by FACS 16 h after the transplantation (n = 8). **b** Migration assay. Freshly purified CD34^+^ cells or cells (2 × 10^5^) expanded ex vivo were cultured in the upper compartment of transwell for 4 h. Total cell migration was quantitated by flow cytometry. Migrated cells toward SDF-1 in the lower chamber significantly increased in hD1R-cultured ones (n = 4). **c** hD1R increased the expression of integrins in CD34^+^ cells. Human CD34^+^ cells were incubated with HUVECs + h5GF in the presence of PBS or hD1R for 7 days. The hematopoietic cells in suspension or Day 0 CD34^+^ cells were collected, and the expression of α4, α6 integrins, and CXCR4 was estimated by using real time RT-PCR (n = 4). Bars = mean ± SD. *P < 0.05

Increased HSC frequency by hD1R could result from HSC number increase or cell-cycle status or improved homing capacity of HSCs. To evaluate the homing efficiency of CD34^+^ cells, we transplanted cultured CD34^+^ cells derived from different conditions into sublethally irradiated NOD/SCID mice. There was no difference in the percentage of hCD45^+^ cells homing to marrow between PBS-cultured cells and Day 0 cells. However, significantly more hCD45^+^ cells homing to the marrow in hD1R-cultured ones (Fig. 4a). Numerous cooperative mechanisms were believed to regulate HSC homing to the marrow, which included adhesion receptor molecules, the stromal cell–derived factor-1 (SDF-1)-CXCR4 and so on. To determine whether the enhanced homing of hD1R-cultured cells was due to up-regulation of SDF-1/CXCR4 signaling, we performed in vitro migration assays and found a significant increase in hD1R-cultured cells migration toward SDF-1 (Fig. 4b). In addition, we tested expression levels of some adhesion receptor molecules and CXCR4 in the cultured cells. The result showed hD1R did not up-regulate CXCR4 expression on the cultured cells, but increased α4 and α6 integrin mRNA levels (Fig. 4c).

### hD1R promoted donor cell engraftment in BM transplantation

**Fig. 5:**
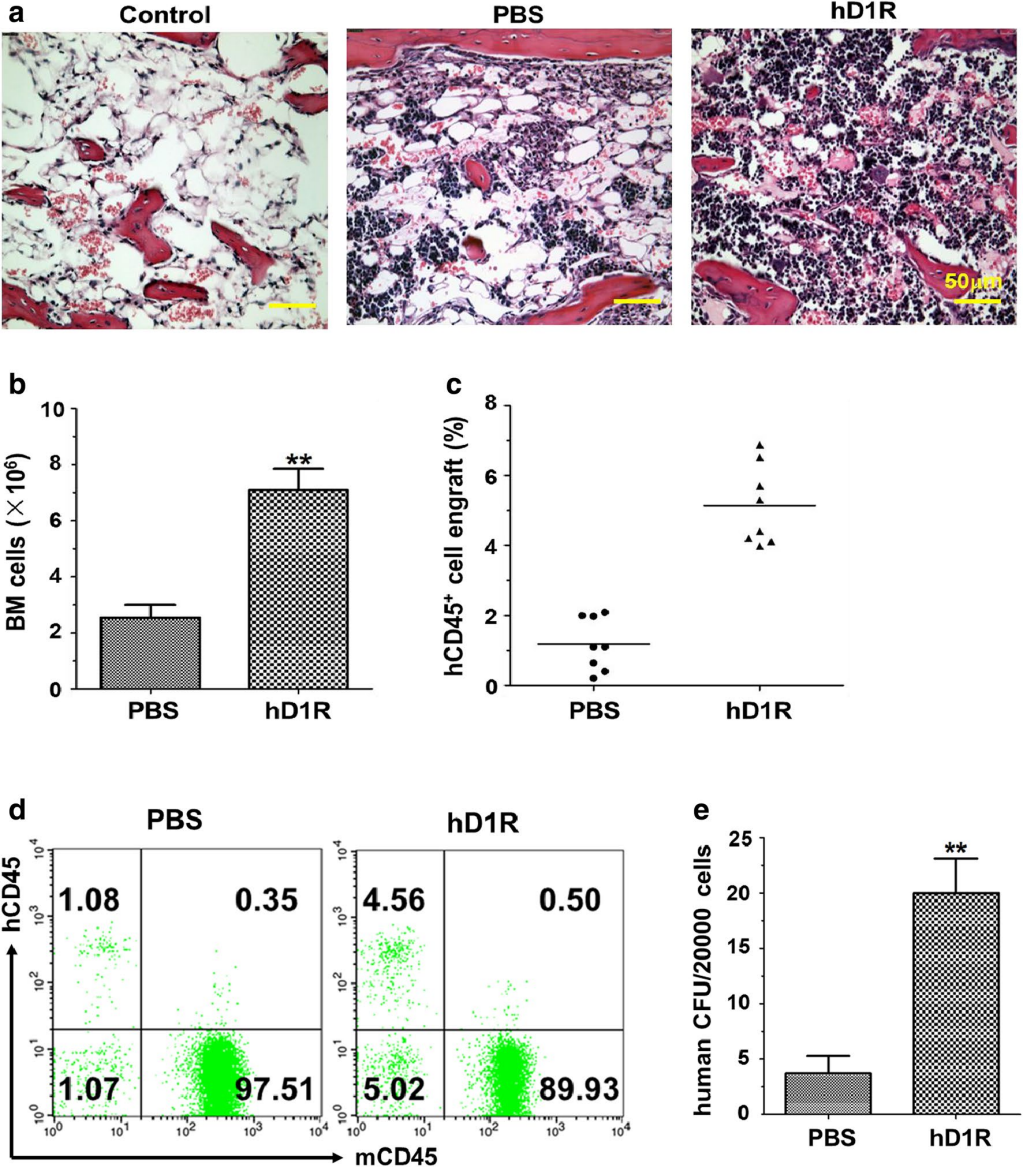
hD1R facilitated donor cell engraftment after BM transplantation. **a** NOD/SCID mice were sublethally irradiated and transplanted with 5 × 10^4^ human UCB CD34^+^ cells. From the day of BM transplantation, mice were daily injected i.p. with PBS or hD1R for 7 days. On day 14 after irradiation, histology of the femurs of mice was examined by using H&E staining. **b** On the 14th day after BM transplantation, BM cells were collected and counted (n = 10). **c** Four weeks after the transplantation, the peripheral blood cells of the recipient mice were analyzed by FACS, and the percentages of engrafted human CD45^+^ cells were shown (n = 8). **d** Representative FACS analysis of CD45^+^ cell engraftment in (**c**). **e** BM cells from the recipient mice in (**a**) were subjected to CFU assay (n = 4). Bars = mean ± SD. *P < 0.05, **P < 0.01

We assessed whether hD1R administration could augment the proliferation of transplanted donor cells in the host. Sublethally irradiated NOD/SCID mice were transfused with the limiting dose of human UCB CD34^+^ cells (5 × 10^4^ cells), and were injected i.p. daily with PBS or hD1R (50 μg) from the day of BM transplantation for 7 days. On day 14 after irradiation, histological examination of BM showed that the structure of BM from the hD1R group showed significantly increased cellularity compared to the PBS or control group (irradiated mice receiving no donor cells) (Fig. 5a). The number of total bone marrow cells in hD1R-treated mice was higher than those in the PBS group (7.09 ± 0.26 × 10^6^ vs. 2.54 ± 0.16 × 10^6^, *P* < 0.01, Fig. 5b). At 4 weeks after transplantation, the percentages of donor cells (hCD45^+^ cells) in peripheral blood were determined by FACS. The hD1R-treated mice significantly increased hCD45^+^ cell repopulation compared with the PBS group (Fig. 5c, d). Colony-forming assay of BM cells showed higher BM CFU counts in hD1R-treated mice than in PBS-treated ones (Fig. 5e). These results showed that hD1R promoted human CB HSPCs regeneration in vivo and accelerated hematopoietic recovery, particularly in a setting where the HSC dose was limiting.

## Discussion

We previously demonstrated a new type of soluble Notch ligand composed of the DSL domain of Dll1 and a RGD motif (D1R) had a strong activity to activate Notch signaling and enhanced HSPCs expansion ex vivo [25, 26]. Herein, we evaluated the in vivo effect of hD1R on hematologic recovery and the engraftment of donor cells in HSCT.

We found that hD1R supplemented HUVEC monolayers and a cytokine combination significantly decreased cell apoptosis and induced UCB HSPCs expansion, which were consistent with our previous study [25]. In addition, exposure to h5GF plus hD1R plus supernatant in the absence of HUEVCs or fixed HUVECs caused a decline in HSPCs. Moreover, hD1R in combination with h5GF plus live HUVECs was able to maintain higher G0/G1 populations of HSPCs, which were capable of rapid multi-lineage reconstitution and maintain long-term SCID-repopulating capacity in serial transplantation. Delaney et al. reported a Delta1^ext-IgG^-mediated ex vivo expansion system for human UCB HSPCs which resulted in a marked increase of HSPCs. When these expanded cells were infused to patients undergoing a myeloablative preparative regimen, the time to neutrophil recovery was significantly reduced. However, longer-term engraftment of donor cells derived from the expanded cells was observed in one of ten patients, suggesting a deficiency of true HSCs expansion and that early myeloid recovery was attributed to short-term repopulating progenitor cells. Of course, we could not exclude this possibility that the expanded cells was rejected by the unmanipulated cells [24]. The function of HSCs might be impaired because the ex vivo culture system deprived HSCs of microenvironmental components [27]. Previous studies demonstrated the vascular niche regulates the survival, proliferation and differentiation of HSCs. Bone marrow sinusoidal ECs played functionally important roles [28]. Butler et al. [13] confirmed the role of the ECs was essential in HSC expansion. Similarly, our results demonstrated live HUVECs were necessary for HSPCs expansion. Moreover, hD1R may promote closer cellular contact between HSCs and HUVECs, which mimiced a physiological microenvironment to improve self-renewal and proliferation of HSCs.

Previous study demonstrated mD1R could directly stimulate hematopoiesis in vivo in normal mice and sublethally irradiated mice [25]. Here, we found that hD1R administration dramatically enhanced the engraftment of donor HSCs after transplantation. Moreover, hD1R significantly boosted the proliferation of BM donor cells and promoted the recovery of the structure and organization of BM microenvironment after irradiation, consistent with the role of Notch signaling in regulating EC proliferation and differentiation [29]. Meanwhile, we continuously monitored the mice injected with hD1R for body weight and hematopoietic parameters until the end of the study. We did not observe tumors occurred, suggesting hD1R may be safe for further application. Many clinical studies showed that the doses of total nucleated cells and CD34^+^ cells in UCBT affected the rate of neutrophil and platelet engraftment, which was closely related with the incidence of transplant-related complication and overall survival [30]. We also found that hD1R substantially shortened the average time of neutrophil and platelet recovery (data not shown). These results demonstrated that hD1R may have a potential to be used in clinical therapy of human diseases such as myelotoxic chemo- or radio-therapy and increase the efficiency of UCBT.

It was reasonable that the rapid engraftment was likely a result of the direct expansion of HSPCs ex vivo and in vivo by hD1R. Moreover, hD1R improved homing capacity of HSCs to facilitate UCB HSPCs engraftment. Studies showed Notch signaling regulated the expression of CXCR4, a critical receptor for HSC homing [31, 32]. Here, hD1R didn’t up-regulate CXCR4 expression on HSPCs. Indeed, the mRNA levels of the α4 and α6 integrin were up-regulated, consistent with the role of adhesion molecules on transplanted HSCs in increasing engraftment [33, 34]. It was possible that Notch signaling crosstalked with other important signaling pathways involved in stem cell niches to help the homing and proliferation of HSCs [35].

## Conclusions

In summary, this study showed that hD1R promoted both hematopoietic recovery and long-term engraftment of human UCB HSPCs. hD1R may represent a new cytokine and have potential clinical utility for HSCT.

## Additional file

10.1186/s12967-015-0761-0 Human UCB CD34^+^ cells expansion by hD1R requires direct cellular interaction with live HUVECs.

## Abbreviations

UCB: umbilical cord blood
HSPC: hematopoietic stem and progenitor cell
DSL: Delta-Serrate-Lag-2
HUVEC: human umbilical vein endothelial cell
LT-HSC: long-term hematopoietic stem cell
NOD/SCID: Nonobese Diabetic-Severe Combined Immunodeficiency
Hes: Hairy and enhancer of split
BM: bone marrow
LB: Luria–Bertani
IPTG: isopropyl β-D-thiogalactoside
FBS: fetal bovine serum
ECGS: endothelial cell growth supplement
PFA: paraformaldehyde
CFU: Colony-forming units
BFU-E: burst-forming unit-erythroid
CFU-GM: CFU-granulocyte macrophage
CFU-GEMM: CFU-granulocyte, erythrocyte, monocyte, macrophage
SRC: SCID-repopulating cell
PB: peripheral blood
