# Supplementary material for: Endothelium-targeted human Delta-like 1 enhances the regeneration and homing of human cord blood stem and progenitor cells
Source: J Transl Med. 2016 Jan 6;14:5. doi: 10.1186/s12967-015-0761-0 (PMC4704259; doi:10.1186/s12967-015-0761-0)
Supplement: Supplementary file 1 — 10.1186/s12967-015-0761-0 Human UCB CD34+ cells expansion by hD1R requires direct cellular interaction with live HUVECs. [file 12967_2015_761_MOESM1_ESM.pdf]

Supplementary materials

Supplementary Figure S1.

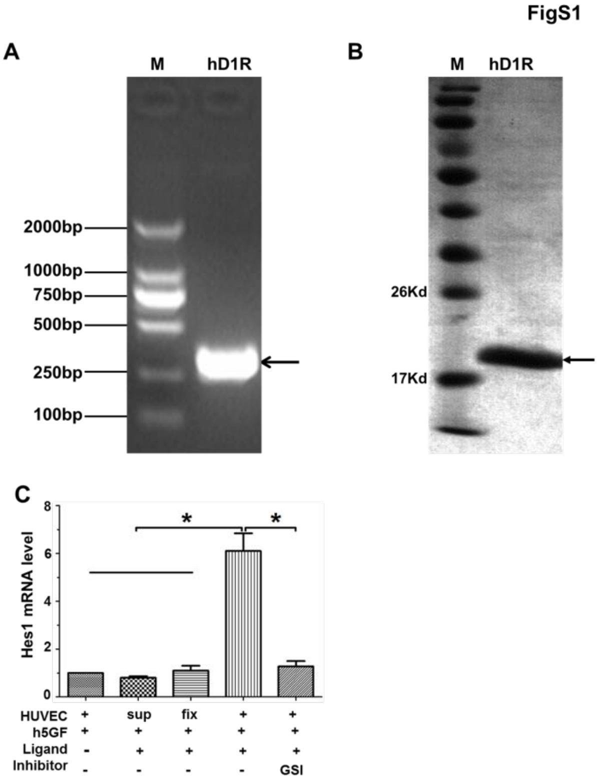

Figure S1. Production of hD1R proteins. The cDNA fragment encoding the DSL domain (amino acids 127-225) of human Dll1 was amplified by PCR using a human cDNA library as a template. The product was fused at the C-terminus with a RGD-encoding fragment (hD1R) by PCR. The hD1R protein was produced and purified as described in Materials and methods. (A) The cDNA fragment encoding hD1R was amplified by PCR indicated by arrows. (B) The recombinant hD1R protein was purified with Ni^{+}-NTA columns and analyzed by using SDS-PAGE. hD1R proteins was detected at the position of 18kd. (C) hD1R bound to live HUVECs and activated Notch signaling. Human UCB $CD34^{+}$ cells were incubated with or without HUVECs in the presence of h5GF plus hD1R for 7 days. GSI was included in some of the cultures. The hematopoietic cells in suspension were collected, and the expression of Hes1 was estimated by using real time RT-PCR (n = 4). Bars = means ± s.d. $^{*}P <$ 0.05.

Supplementary Figure S2.

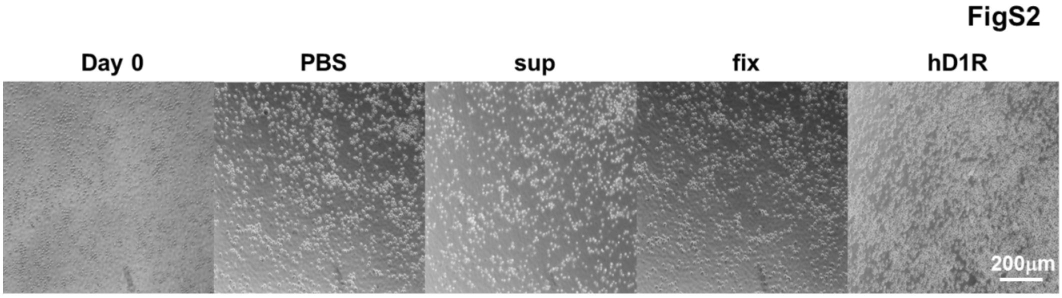

Figure S2. hD1R expanded human UCB CD34$^+$ cells. Human UCB CD34$^+$ cells were cultured with or without HUVECs in the presence of h5GF plus hD1R for 7 days. Representative phase-contrast micrographs of expanded hematopoietic cells were shown in different culture condition at day 7.

Supplementary Figure S3.

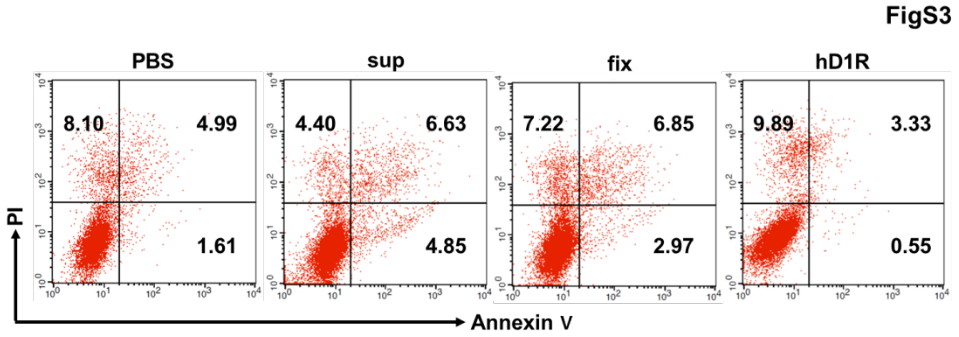

Figure S3. hD1R inhibited cell apoptosis in the ex vivo expansion system. Human UCB CD34$^+$ cells were cultured with or without HUVECs in the presence of h5GF plus hD1R for 7 days. Cell apoptosis was determined by using Annexin V staining followed by FACS. A representative FACS analysis plot was shown.

Supplementary Table S1.

Table S1 Limiting dilution analysis of SRC frequency

Independent experiments	$CD34^+$ starting cell dose per mouse	Number of mice engrafted with ≥0.5% human $CD45^+$ cells per total number of mice		
		Day 0	PBS	hD1R
Expt.1	1000	0 of 10	0 of 10	1 of 10
	6000	1 of 10	1 of 10	3 of 10
	10000	2 0f 10	3 0f 10	10 0f 10
	Frequency of SRCs	1 in 52208	1 in 37815	1 in 6976
	95% confidence interval	1/161512 - 1/16876	1/100325 - 1/14253	1/11965 - 1/4067
Expt.2	1000	0 of 10	0 of 10	2 of 10
	6000	1 of 10	2 of 10	6 of 10
	10000	2 0f 10	4 0f 10	10 0f 10
	Frequency of SRCs	1 in 52208	1 in 23724	1 in 4422
	95% confidence interval	1/161512 - 1/16876	1/52723 - 1/10676	1/7419 - 1/2636
Expt.3	1000	0 of 10	1 of 10	1 of 10
	6000	1 of 10	3 of 10	4 of 10
	10000	3 0f 10	6 0f 10	10 0f 10
	Frequency of SRCs	1 in 37815	1 in 12585	1 in 6176
	95% confidence interval	1/100325 - 1/14253	1/12585 - 1/6704	1/10484 - 1/3638

Table S1. Limiting dilution analysis of SCID-repopulating cell frequency.

$CD34^+$ cells freshly purified from UCB (Day 0) (10^3, 6×10^3, 10×10^3 starting cells) or their progeny expanded with HUVECs + h5GF plus PBS or hD1R were transplanted into sublethally irradiated NOD/SCID mice. Overall human cells engraftment data were shown in three independent experiments. Mice were considered engrafted if they had ≥0.5% human $CD45^+$ cells in the peripheral blood at 12 weeks post transplantation.
